# Perception and attitudes of medical students on clinical clerkship in the era of the Coronavirus Disease 2019 pandemic

**DOI:** 10.1080/10872981.2020.1809929

**Published:** 2020-08-25

**Authors:** So Mi Kim, Seok Gun Park, Young Koo Jee, Il Han Song

**Affiliations:** a Departments of Internal Medicine, Dankook University College of Medicine, Dankook University Hospital, Republic of Korea; b Department of NuclearMedicine, Dankook University College of Medicine, Dankook University Hospital, Republic of Korea

**Keywords:** COVID-19, clinical clerkship, medical students, medical education, perception, attitudes

## Abstract

**Background:**

The Coronavirus Disease 2019 (COVID-19) has been placing severe strain on global healthcare systems and medical education programs, leading to growing demands for medical students to assume the role of preliminary healthcare providers.

**Objectives:**

To assess the perception and attitudes of medical students about clinical clerkship training during the COVID-19 pandemic.

**Design:**

A cross-sectional survey with web-based 3-fields/14-items questionnaire was conducted, from April 7 to 14, 2020, to evaluate their self-assessed perception and attitudes on clerkship training of hospital practice under the COVID-19 outbreak and spread among 161 (78 on pre-clerkship course, 83 on clinical clerkship course) medical students at Dankook University College of Medicine, Cheonan, Republic of Korea.

**Results:**

Of the 151 medical students who completed the survey, 81 students (53.7%) considered themselves familiar with COVID-19. Although the students were concerned about the spread of the virus during clinical clerkship training, 118 (78.1%) students preferred the clerkship training in a hospital practice. The students in the clinical clerkship program preferred this over those in the pre-clerkship program (85.7% vs. 70.2%, *P* = 0.03), primarily because a clinical clerkship could not be replaced by an online class during the COVID-19 pandemic. In addition, their responses indicated, in order of significance, fear of not completing the clerkship course on time, willingness to participate as a preliminary healthcare provider in pandemic, the potential waste of tuition, and belief that a hospital is rather safe. The change in the academic calendar had not a positive impact on the lifestyles of many students.

**Conclusions:**

In circumstances such as the COVID-19 pandemic, educational strategies to clinical clerkship training for medical students should be developed to provide them with the opportunity to be actively involved in hospital practice under strict safety guidance focused on preventing virus infection and transmission.

## Introduction

The Coronavirus Disease 2019 (COVID-19) is currently placing huge pressure on the medical community around the world. Since the outbreak of this novel infectious disease by severe acute respiratory syndrome-coronavirus-2 (SARS-CoV-2) in Wuhan, China, in late 2019, the status of the disease has changed from ‘epidemic’ to ‘pandemic,’ putting countries on high alert to this menace to their national public health [1–3]. Many countries have been devoting all available resources to providing medical healthcare services for the treatment of COVID-19-positive individuals, preventing the spread of the virus and community infection, and enacting quarantine-based official restrictions on social activity [4]. However, despite countries’ intensive effort to install these healthcare measures, it is unrealistic to expect a reduction in the virus itself or a reduction in the increased healthcare burden until the emergence of an approved antiviral treatment regimen and the development of vaccine against COVID-19 [5,6]. Where this pandemic is likely to continue, countries’ healthcare systems may struggle to cope owing to a shortage of healthcare personnel, medical facilities, and/or equipment.

Recently, the availability of hospital-based roles for medical students during the COVID-19 pandemic has been recognized as an important issue [7–9]. Rescheduling the medical academic calendar has an important bearing on the current status of the COVID-19 pandemic. The Korean Association of Medical Colleges (KAMC) has strongly recommended the postponement of the start of the educational calendar for several weeks, and the transition to remote learning with online lectures over direct clerkship training within a hospital, to all medical colleges. The Association of American Medical Colleges (AAMC) has also instructed medical schools to suspend student clerkships [10–12]. Due to the distinctive characteristics of the medical college curriculum, KAMC subsequently stated that medical colleges could reschedule and become more flexible to enable the education of students engaged in clerkship programs at training hospitals while adhering to safety guidance regarding COVID-19 infection and transmission. Most medical colleges have followed the KAMC’s guidance [13]. These recommendations seem to be reasonable, as they place the priority on student safety during the COVID-19 outbreak; however, it is considered that they do not sufficiently reflect medical students’ own opinions about clinical clerkship programs. It is important to understand the concerns and attitudes of medical students surrounding clerkship training in a hospital setting under circumstances such as the COVID-19 pandemic. In addition, educational policies such as the development of alternative clerkship programs, new modes of student–patient interaction, and the re-establishment of medical students’ roles are vital issues to consider. This study aimed to assess the perception and attitudes of medical students about these changes to clinical clerkship training programs in South Korea during the COVID-19 pandemic.

## Methods

### Participants

We enrolled a total of 161 medical students at Dankook University College of Medicine in this web-based, cross-sectional survey. The students represented four levels of training: 41 were in the first year of medical college, 37 in the second year, 44 in the third year, and 39 students were in the fourth year. Due to the outbreak of COVID-19, the first day of the new semester was postponed for a few weeks, so it began later than was originally scheduled on the academic calendar. The Dankook University authorities deemed it necessary to replace all face-to-face classes with online classes for students in the pre-clerkship stage (first year and second year); clinical clerkship training proceeded as usual for students in the clerkship stage (third year and fourth year). We conducted this survey over 2 weeks between April 7 and 21, 2020, during the 2 weeks following the start of the delayed semester. Due to the restrictions imposed by the COVID-19 pandemic, face-to-face surveys were not possible. Hence, all students were contacted by phone from the Administrative team of Dankook University College of Medicine and asked to participate using an online Google Docs survey. The link for the survey was informed by a text message. The principles of voluntary participation, no harm to the participants, anonymity and confidential assurances were announced at the beginning of the questionnaire.

### Study design and data collection

We developed the questionnaires to evaluate medical students’ thoughts on clinical clerkship training during the COVID-19 pandemic from the viewpoints of both preliminary healthcare providers and university students. We also prepared both Korean- and English-language versions of the questionnaire to enable the inclusion of international students in the survey, and conducted a pilot testing among six students: three pre-clerkship and three clinical clerkship students, with each group including one international student to reduce the likelihood of questions that could lead to misunderstandings or biased answers from participants. The study questionnaires were composed of the following three fields: 1) students’ knowledge and perception of COVID-19 (three questions); 2) students’ attitudes toward clinical clerkship training during the COVID-19 pandemic (six questions); and 3) the impact of the change in the academic calendar due to the COVID-19 pandemic on students’ routines and lifestyles (four questions). Enrolled students completely answered the questions addressing their own knowledge of COVID-19 based on self-assessment, and the questions about risk perception on the likelihood of contracting the novel coronavirus during the clinical clerkship training. The questions addressing students’ attitudes toward clinical clerkship training during the COVID-19 pandemic measured their willingness to participate and concerns regarding qualitative changes in the clinical clerkship. Furthermore, we evaluated their answers to questions about the impact of the changes to the academic calendar on students’ lifestyles by considering both the magnitude and significance of these lifestyle changes and the effectiveness of online classes at home. Most of the questionnaires consisted of closed-ended questions to facilitate categorization of the participants’ answers. Dichotomous questions were scored using a two-point scale, and questions with more than two options were scored based on a five-point Likert scale. Open-ended questions were also used to invite a variety of opinions from participants. The present study was approved by the Institutional Review Board of Dankook University Hospital (DKUH 2020–04-054-HE001).

### Statistical analysis

We displayed all data in raw numbers with percentages and estimated the mean and standard deviation values. We analyzed, between two groups, continuous variables by the unpaired student-t-test and categorical variables by the χ2 test or Fisher’s exact test. All analyses were performed using SPSS version 25 software for Windows (SPSS Inc., Chicago, IL, USA). Statistical signiﬁcance was determined at *P* < 0.05.

## Results

### Baseline characteristics of participants

Among the 161 medical students, 151 (93.8%) completed the survey. The baseline characteristics of participants are summarized in Table 1. The 151 medical students who completed the survey were divided into two groups depending on whether they had entered the clinical clerkship course. Thus, there were 74 students in the pre-clerkship group (first and second years) and 77 in the clinical clerkship group (third and fourth years). There were no differences in gender or ethnicity between the two groups; the mean age was higher in the clinical clerkship than in the pre-clerkship group (24.3 ± 1.3 vs. 22.2 ± 1.7 years, *P* = 0.001).

**Table 1.** Characteristics of participants and their self-assessed knowledge and perceptions about the COVID-19.

**Demographics and survey questionnaires**	**Total students** **(n = 151)**	**Pre-clerkship** **(n = 74)**	**Clinical clerkship (n = 77)**	*P-value
**Age, years±SD**	23.3±1.8	22.2±1.7	24.3±1.3	0.001
**Male gender, n (%)**	101 (66.9)	55 (74.3)	50 (64.9)	0.222
**Ethnicity**				0.06
Korean, n (%)	144 (95.4)	73 (98.6)	71 (92.2)	
Arabian, n (%)	7 (4.6)	1(1.4)	6 (7.8)	
**Years**				-
1st/2nd, n (%)	41/33 (27.2/21.9)	41/33 (55.4/44.6)	-	
3rd/4th, n (%)	40/37 (26.5/24.5)	-	40/37 (51.9/48.1)	
**QA 1. How much do you think you know about the COVID-19?** 0.573				
Far below average	5 (3.3)	2 (2.7)	3 (3.9)	
Below average	9 (6.0)	4 (5.4)	5 (6.5)	
Average	56 (37.0)	32 (43.2)	24 (31.2)	
Above average	72 (47.7)	33 (44.6)	39 (50.6)	
Far above average	9 (6.0)	3 (4.1)	6 (7.8)	
**QA 2. Do you think SARS-CoV-2 can spread from patients to students during clinical clerkship course?** 0.086				
Definitely not	8 (5.3)	6 (8.1)	2 (2.6)	
Probably not	31 (20.5)	11 (14.9)	20 (25.9)	
Unsure	30 (19.9)	12 (16.2)	18 (23.4)	
Probably yes	63 (41.7)	37 (50)	26 (33.8)	
Definitely yes	19 (12.6)	8 (10.8)	11 (14.3)	
**QA 3. Do you think SARS-CoV-2 can spread from students to patients during clinical clerkship course?** 0.129				
Definitely not	3 (2.0)	3 (4.1)	0 (0)	
Probably not	28 (18.5)	13 (17.6)	15 (19.5)	
Unsure	43 (28.5)	20 (27.0)	23 (29.9)	
Probably yes	62 (41.1)	34 (45.9)	28 (36.4)	
Definitely yes	15 (9.9)	4 (5.4)	11 (14.2)	

Note: * *P*-value was calculated between the students on pre-clerkship course and on clinical clerkship course. COVID-19: coronavirus disease 2019, *SARS-CoV-2*: severe acute respiratory syndrome-coronavirus-2, QA: questionnaire A, SD: standard deviation.

### Students’ self-assessed knowledge and perceptions about COVID-19

More than half of these medical students (53.7%) believed that their knowledge of COVID-19 was ‘above average’ or ‘far above average’; there was little difference between the pre-clerkship and clinical clerkship groups (48.7% vs. 58.4%). During the clerkship training, students’ perception of the SARS-CoV-2 transmissibility was higher in the pre-clerkship than in the clerkship group, but not significantly so (transmission possible from patients to students, 60.8% vs. 48.1%, *P* = 0.086; transmission possible from students to patients, 51.3% vs. 50.6%, *P* = 0.129) (Table 1).

### Students’ attitudes toward clinical clerkship training during the COVID-19 pandemic

**Figure 1.**
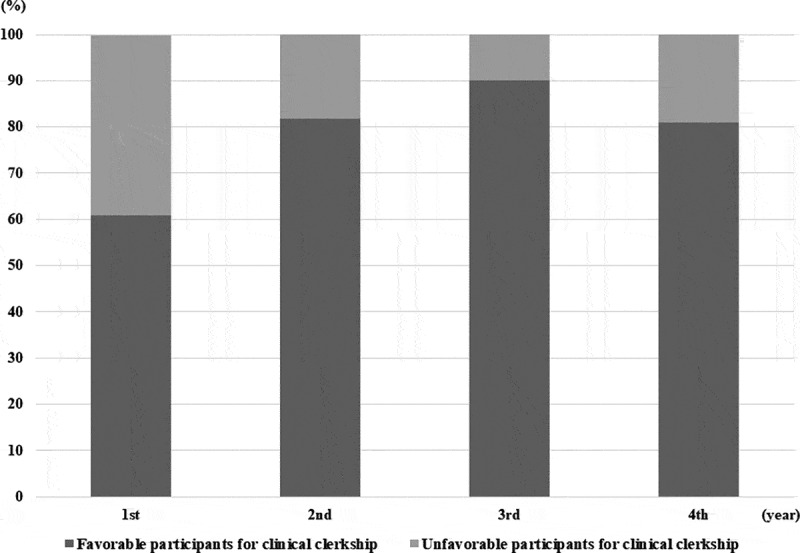
Year distribution of medical students (N = 151) according to preference of participation in clinical clerkship training in COVID-19 pandemic. COVID-19: coronavirus disease 2019.

**Figure 2.**
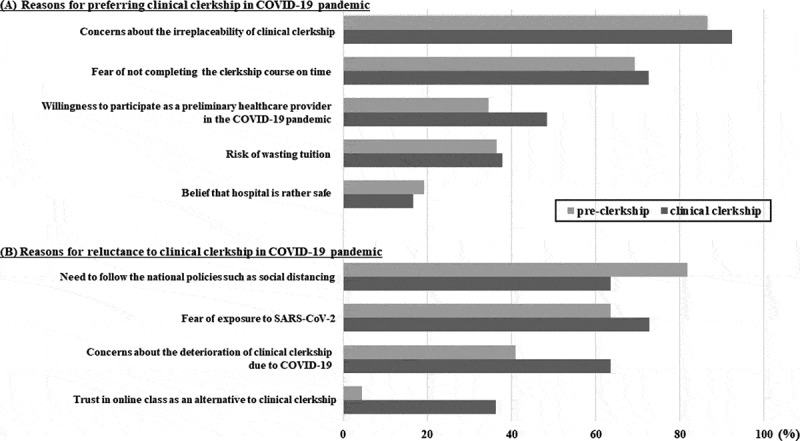
Reasons of determining the preference of medical students (N = 151) for clinical clerkship training in COVID-19 pandemic. (A) Reasons for preferring clinical clerkship, (B) Reasons for reluctance to clinical clerkship. COVID-19: coronavirus disease 2019, SARS-CoV-2: severe acute respiratory syndrome-coronavirus-2.

The students’ attitudes toward clinical clerkship training during the COVID-19 pandemic are summarized in Table 2. Twenty-seven (17.9%) students were not satisfied with the postponement of the academic calendar due to the COVID-19 pandemic. Although the students were concerned that the virus could spread during clinical clerkship training, 118 (78.1%) students believed that they should take the clerkship training in hospital practice, nonetheless. This preference was greater among students in the clinical clerkship course than those in the pre-clerkship course (85.7% vs. 70.2%, *P* = 0.03). Of particular note, the willingness to participate in the clinical clerkship course was stronger in students in the third year than those in the fourth year (Figure 1). Students felt they should take the clerkship training during the COVID-19 pandemic for the following reasons, in order of significance: concerns that an online class could not replace the clinical clerkship course (86.5% of students on pre-clerkship vs. 92.4% of students on clerkship); a fear of not completing the clerkship course on time (69.2% vs. 72.5%, respectively); a willingness to participate as a preliminary healthcare provider (34.6% vs. 48.5%, respectively); the risk of wasting tuition (36.5% vs. 37.8%, respectively), and the belief that a hospital is rather safe (19.2% vs. 16.6%, respectively). The differences between the two groups were not statistically significant (*P* = 0.441). In contrast, where students in the pre-clerkship and clinical clerkship groups were reluctant to participate in the clinical clerkship, the reasons were as follows: a need to follow national policies such as social distancing (81.8% vs. 63.6%, respectively); a fear of exposure to SARS-CoV-2 (63.6% vs. 72.7%, respectively); concerns about the deterioration of the clinical clerkship due to the spread of COVID-19 (40.9% vs. 63.6%, respectively); and a trust in online classes as a viable alternative to the clinical clerkship (4.5% vs. 36.3%, respectively). These differences were statistically significant (*P* = 0.041) (Figure 2). Approximately one-third of students thought the quality of clinical clerkship training would be reduced due to limitations imposed on various clinical experiences by the COVID-19 pandemic.

**Table 2.** Attitudes of participants toward clinical clerkship training in the COVID-19 pandemic.

Survey questionnaires	Total students (n = 151)	Pre-clerkship (n = 74)	Clinical clerkship (n = 77)	*P-value
**QB 1. Have you been notified by Medical College about a change in academic calendar due to COVID-19?**				
Yes, n (%)	107 (70.8)	51 (68.9)	56 (72.7)	0.6
**QB 2. Are you satisfied with changed academic calendar due to COVID-19?** 0.112				
Very dissatisfied	8 (5.3)	4 (5.4)	4 (5.2)	
Dissatisfied	19 (12.6)	8 (10.8)	11 (14.3)	
Unsure	51 (33.8)	21 (28.4)	30 (39.0)	
Satisfied	40 (26.5)	18 (24.3)	22 (28.5)	
Very satisfied	33 (21.8)	23 (31.1)	10 (13.0)	
**QB 3. Do you think you should participate in clinical clerkship training as scheduled in spite of the COVID-19 pandemic?**				
Yes, n (%)	118 (78.1)	52 (70.2)	66 (85.7)	0.03
**QB 4. What are your family’s concerns about your clinical clerkship training in the COVID-19 pandemic?** 0.002				
Not at all concerned	13 (8.6)	6 (8.1)	7 (9.0)	
Slightly concerned	49 (32.5)	16 (21.6)	33 (42.9)	
Somewhat concerned	39 (25.8)	29 (39.2)	10 (13.0)	
Moderately concerned	35 (23.2)	18 (24.3)	17 (22.1)	
Extremely concerned	15 (9.9)	5 (6.8)	10 (13.0)	
**QB 5. Do you think the quality of clinical clerkship training will be reduced by the COVID-19 pandemic?** 0.212				
Definitely not	4 (2.6)	1 (1.4)	3 (3.9)	
Probably not	28 (18.6)	9 (12.2)	19 (24.7)	
Unsure	65 (43.1)	34 (45.9)	31 (40.2)	
Probably yes	47 (31.1)	27 (36.5)	20 (26.0)	
Definitely yes	7 (4.6)	3 (4.0)	4 (5.2)	
**QB 6. Do you think you may have various experiences in clinical clerkship training during the COVID-19 pandemic?** 0.341				
Definitely not	14 (9.3)	4 (5.4)	10 (13.0)	
Probably not	48 (31.8)	21 (28.4)	27 (35.1)	
Unsure	69 (45.7)	37 (50.0)	32 (41.5)	
Probably yes	18 (11.9)	11 (14.9)	7 (9.1)	
Definitely yes	2 (1.3)	1 (1.3)	1 (1.3)	

Note: * *P*-value was calculated between the students on pre-clerkship course and on clinical clerkship course. COVID-19: coronavirus disease 2019, QB: questionnaire B.

### Impact of changes in the academic calendar on students’ lifestyle during the COVID-19 pandemic

More than two thirds (69.5%) of the 151 medical students surveyed believed that the postponed academic calendar had a significant impact on their way of life. This belief was stronger, but not significantly so, for students in the pre-clerkship than those in the clinical clerkship course (75.7% vs. 63.6%). Although students generally believed the postponed academic calendar had a negative impact on their lifestyle, the students in the pre-clerkship course felt more positively about the situation (35.1% vs. 9.1%, *P* = 0.002). Lifestyle factors affected by the change of the academic calendar included sleep habits (58.9%), physical activity (54.3%), increased stress on academic achievement (43.0%), dietary patterns (42.4%), and alcohol consumption (15.2%). The majority of students believed that in cases where the clinical clerkship training was changed to an online class at home, participation in or understanding of the class would be significantly reduced (Table 3).

**Table 3.** Impact of a change in academic calendar on lifestyle in the COVID-19 pandemic.

Survey questionnaires	Total student (n = 151)	Pre-clerkship (n = 74)	Clinical clerkship (n = 77)	*P-value
**QC 1. How much did a change in the academic calendar affect your lifestyle by COVID-19 pandemic?** 0.266				
Insignificant	4 (2.6)	2 (2.7)	2 (2.6)	
Minor	17 (11.3)	4 (5.4)	13 (16.9)	
Moderate	25 (16.6)	12 (16.2)	13 (16.9)	
Major	64 (42.4)	34 (46.0)	30 (39.0)	
Severe	41 (27.1)	22 (29.7)	19 (24.6)	
**QC 2. How significant did a change in the academic calendar affect your lifestyle by COVID-19 pandemic?** 0.002				
Very negative	9 (6.0)	5 (6.8)	4 (5.2)	
Negative	54 (35.8)	24 (32.4)	30 (38.9)	
Neutral	55 (36.4)	19 (25.7)	36 (46.8)	
Positive	22 (14.5)	17 (23.0)	5 (6.5)	
Very positive	11 (7.3)	9 (12.1)	2 (2.6)	
**QC 3. Do you think class participation will increase, if the current class is replaced with an online class at home?** 0.070				
Definitely not	34 (22.5)	11 (14.9)	23 (29.8)	
Probably not	58 (38.4)	29 (39.2)	29 (37.7)	
Unsure	49 (32.5)	27 (36.5)	22 (28.6)	
Probably yes	9 (6.0)	7 (9.4)	2 (2.6)	
Definitely yes	1 (0.6)	0 (0)	1 (1.3)	
**QC 4. Do you think the class will be better understood, if the current class is replaced with an online class at home?** 0.167				
Definitely not	37 (24.5)	12 (16.2)	25 (32.5)	
Probably not	65 (43.0))	35 (47.3)	30 (39.0)	
Unsure	37 (24.5)	20 (27.0)	17 (22.0)	
Probably yes	9 (6.0)	6 (8.1)	3 (3.9)	
Definitely yes	3 (2.0)	1 (1.4)	2 (2.6)	

Note: * *P*-value was calculated between the students on pre-clerkship course and on clinical clerkship course. COVID-19: coronavirus disease 2019, QC: questionnaire C.

## Discussion

In this cross-sectional analysis, most medical students, especially third-year students, despite the COVID-19 pandemic, showed a commitment to clinical clerkship training with the thought that it can not be replaced with any class and that they have to participate as a preliminary healthcare provider. In the past, such as during the outbreaks of Severe Acute Respiratory Syndrome (SARS) caused by the SARS-associated coronavirus in 2003 and of Middle East Respiratory Syndrome (MERS) caused by MERS coronavirus in 2015, measures of such changes to the academic schedule or partial school closures were implemented, but these were temporary and localized [14,15]. The current global outbreak of COVID-19, however, is even more serious, disrupting educational systems worldwide [16,17]. As the pandemic period of COVID-19 has continued, university authorities have postponed academic calendars and recommended alternative education programs. However, these policies, such as those implemented by the National Ministry of Education, are focused on students’ safety and the prevention of COVID-19 transmission, and they fail to consider the specific qualities of medical education courses and the attitudes of students toward clinical clerkship. Therefore, the results of this study may suggest factors that university authorities should prioritize when making policy decisions for medical education in hospital practice under these circumstances.

In this survey, the majority of medical students showed a positive attitude toward hospital practices at this time, despite concerns that clerkship training during the COVID-19 pandemic could spread the virus. This demonstrates how important it is for students to complete the educational curriculum in a timely manner, as clinical clerkship training is irreplaceable. Additionally, it was possible to reaffirm their identities as doctors through their intention to take part in the COVID-19 crisis as a preliminary healthcare provider, not just as a student. It is particularly interesting to note that third-year students were most likely to express an interest in participating in clinical clerkship training, reaching 90%. In the Korean medical education curriculum, the third year is an introductory period for hospital practice through the clinical clerkship course. It is a difficult time for students in training due to the high intensity of coursework in major subjects, including internal medicine, general surgery, pediatrics, psychiatry, and obstetrics [18]. Therefore, it is considered that curiosity about introductory clinical practice, along with the pressure of the extensive practical education, influenced the higher preference of third-year medical students to participate in clinical clerkship training compared to other-year students. Although a minority of students were reluctant to participate in clinical clerkship training, this was primarily due to their belief that clerkship training during the COVID-19 outbreak was against national policies such as social distancing, rather than a fear of exposure to SARS-CoV-2 or a concern about the deterioration of clinical clerkship. These responses show that students support the current Korean National Policy to prevent the spread of COVID-19 and take this view as citizens, not as medical students. Furthermore, they might feel guilty or confused about participating in clinical clerkship, as it seems to conflict with national policies that are being applied to other non-medical students to assure that they do not attend classes in person. Therefore, these results suggest that university authorities should provide sufficient explanation of the purpose and necessity of the clinical clerkship and ensure that students understand and are able to voice their opinions. They should also ensure that students are not placed in a situation of emotional conflict between the desire to do their clinical clerkship and the requirements of national policy.

During the COVID-19 pandemic, concerns about SARS-CoV-2 transmission from patients to students on clerkship and the reduced quality of clinical clerkship training appeared to be greater in the pre-clerkship group than in the clinical clerkship group. These results are likely due to the fact that students on the pre-clerkship course have had no opportunity to experience various clinical strategies for infection prevention and control within the hospital. However, the expectation of experiencing various clinical practices through the clinical clerkship during the COVID-19 pandemic was lower in the clinical clerkship group compared to pre-clerkship group. Students seemed to feel that restrictions on access to patients and on the use of hospital facilities or equipment resulting from changes in the hospital system designed to prevent the spread of SARS-CoV-2 were not suitable for proper education of students. Therefore, it is necessary to develop revised clinical clerkship programs that allow students to be involved in various medical scenarios that can still be experienced during the COVID-19 pandemic. In addition, these policies should encourage active participation in clinical clerkship training under the strict control of strengthened safety systems.

In this study, a majority of medical students responded that their lifestyles were negatively affected by the postponement of the academic calendar due to COVID-19. With regard to the significance of that impact, students’ routine lifestyle was influenced more positively in the pre-clerkship group than the clinical clerkship group. The results demonstrate that although all face-to-face classes were replaced with online classes at home for the pre-clerkship group, the changes to the academic schedule did not have a great influence on their day-to-day routine. Conversely, even if clerkship training proceeded as usual, there would be many restrictions on social activities outside of training due to COVID-19. If the current academic schedule were to be replaced with online classes, students generally felt that their ability to participate and understand the classes would be poor. However, previous studies have shown that online classes were as useful, effective, and even as enjoyable as traditional didactics [19,20]. Therefore, in preparation for situations such as the COVID-19 pandemic, it is necessary to develop various learning techniques to replace face-to-face classes that can maintain and encourage students’ participation and understanding during non-face-to-face education.

### Limitations

This study has several limitations. First, as a survey study, it depended on participants’ own subjective, rather than objective, views through self-assessment and self-reporting. However, on the other hand, it also demonstrated participants’ willingness and ability to be involved during these difficult circumstances. Second, this survey was conducted following the university’s decision regarding the clinical clerkship training of medical students; thus, there may have been a timing bias as students expressed their response to the hospital practice. However, this may also provide an opportunity to record their feelings while doing their own hospital practice. In addition, given that medical students taking this survey were selected from one university and the number of participants was relatively small, the generalizability of these findings to other medical students in Korea is limited.

## Conclusions

In conclusion, in the era of the COVID-19 pandemic, most medical students have a feeling of confidence in their understanding of COVID-19 and are concerned about its transmission to both themselves and patients during clinical clerkship training in hospitals. Nevertheless, they feel that clinical clerkship is necessary due to the difficulty of replacing hospital clerkships with online lectures, the need for timely achievement of academic goals with minimal disruption, and the desire for participation as required in clinical fields as a preliminary healthcare provider. Educational strategies to clinical clerkship training for medical students should be developed that provide the opportunity for active involvement in practical hospital training under strict safety guidance to prevent virus transmission.

## Supplementary Material

Supplemental Material
