# Supplementary material for: Perception and attitudes of medical students on clinical clerkship in the era of the Coronavirus Disease 2019 pandemic
Source: Med Educ Online. 2020 Aug 25;25(1):1809929. doi: 10.1080/10872981.2020.1809929 (PMC7482770; doi:10.1080/10872981.2020.1809929)
Supplement: Supplemental Material [file ZMEO_A_1809929_SM8780.docx]

**Medical students’ opinion about the clinical clerkship in COVID-19**

**Survey Questionnaires (April 2, 2020)**

| **Personal information** | |
| --- | --- |
| Grade: | Age (in years): |
| Gender: 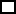 Male 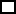 Female | Ethnicity: |

| **PART 1. Students’ self-assessed knowledge and perceptions about COVID-19** | | | | |
| --- | --- | --- | --- | --- |
| 1. How much do you think you know about the COVID-19? | | | | |
| 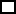 Far below average | 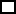 Below average | 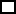 Average | 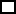 Above average | 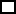 Far above average |
| 2. Do you think SARS-CoV-2 can spread from patients to students during clinical clerkship course? | | | | |
| 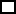 Definitely not | 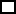 Probably not | 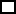 Unsure | 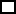 Probably yes | 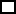 Definitely yes |
| 3. Do you think SARS-CoV-2 can spread from students to patients during clinical clerkship course? | | | | |
| 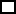 Definitely not | 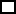 Probably not | 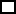 Unsure | 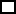 Probably yes | 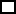 Definitely yes |

| **PART 2. The attitudes of participants toward clinical clerkship training in the COVID-19 pandemic** | | | | |
| --- | --- | --- | --- | --- |
| 1. Have you been notified by Medical College about a change in academic calendar due to COVID-19? | | | | |
| 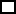 yes | | 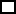 No | | |
| 2. Are you satisfied with changed academic calendar due to COVID-19? | | | | |
| 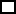 Very dissatisfied | 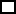 Dissatisfied | 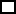 Unsure | 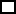 Satisfied | 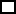 Very satisfied |
| 3. Do you think you should participate in clinical clerkship training as scheduled in spite of the COVID-19 pandemic? | | | | |
| 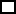 yes | | 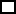 No | | |
| 3-1. If you answer ‘yes’, what are the reasons? (multiple responses allowed) | | | | |
| 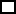 Concerns about the irreplaceability of clinical clerkship | | | | |
| 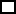 Fear of not completing the clerkship course on time | | | | |
| 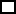 Willingness to participate as a preliminary healthcare provider in the COVID-19 pandemic | | | | |
| 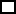 Risk of wasting tuition | | | | |
| 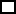 Belief that hospital is rather safe | | | | |
| 3-2. If you answer ‘no’, what are the reasons? (multiple responses allowed) | | | | |
| 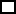 Need to follow the national policies such as social distancing | | | | |
| 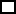 Fear of exposure to SARS-CoV-2 | | | | |
| 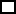 Concerns about the deterioration of clinical clerkship due to COVID-19 | | | | |
| 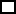 Trust in online class as an alternative to clinical clerkship | | | | |
| 4. What are your family’s concerns about your clinical clerkship training in the COVID-19 pandemic? | | | | |
| 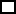 Not at all concerned | Slightly 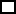 concerned | 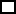 Somewhat concerned | 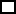 Moderately concerned | 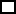 Extremely concerned |
| 5. Do you think the quality of clinical clerkship training will be reduced by the COVID-19 pandemic? | | | | |
| 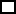 Definitely not | 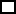 Probably not | 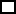 Unsure | 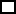 Probably yes | 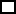 Definitely yes |
| 6. Do you think you may have various experiences in clinical clerkship training during the COVID-19 pandemic? | | | | |
| 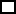 Definitely not | 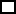 Probably not | 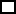 Unsure | 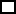 Probably yes | 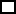 Definitely yes |

| **PART 3. The impact of a change in academic calendar on lifestyle in the COVID-19 pandemic** | | | | |
| --- | --- | --- | --- | --- |
| 1. How much did a change in the academic calendar affect your lifestyle by COVID-19 pandemic? | | | | |
| 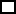 Insignificant | 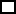 Minor | 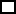 Moderate | 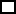 Major | 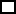 Severe |
| 2. How significant did a change in the academic calendar affect your lifestyle by COVID-19 pandemic? | | | | |
| 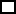 Very negative | 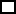 Negative | 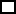 Neutral | 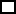 Positive | 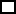 Very positive |
| 3. What are your lifestyle factors that have affected by the change of the academic calendar due to COVID-19?  (multiple responses allowed) | | | | |
| 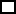 Dietary patterns | 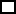 Sleep habits | 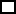 Physical activity | 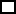 Stress on academic achievement | |
| 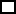 Alcohol consumption | |  |  |  |
| 4. Do you think class participation will increase, if the current class is replaced with an online class at home? | | | | |
| 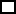 Definitely not | 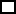 Probably not | 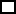 Unsure | 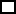 Probably yes | 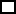 Definitely yes |
| 5. Do you think the class will be better understood, if the current class is replaced with an online class at home? | | | | |
| 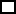 Definitely not | 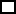 Probably not | 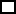 Unsure | 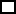 Probably yes | 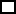 Definitely yes |
